# The cost of entry: An analysis of pharmaceutical registration fees in low-, middle-, and high-income countries

**DOI:** 10.1371/journal.pone.0182742

**Published:** 2017-08-15

**Authors:** Steven G. Morgan, Brandon Yau, Murray M. Lumpkin

**Affiliations:** 1 School of Population and Public Health, Faculty of Medicine, University of British Columbia, Vancouver, British Columbia, Canada; 2 Faculty of Medicine, University of British Columbia, Vancouver, British Columbia, Canada; 3 Bill & Melinda Gates Foundation, Seattle, Washington, United States of America; UNAIDS, GUYANA

## Abstract

**Background:**

Advances in pharmaceuticals offer improved health outcomes for a wide range of illnesses, yet medicines are often inaccessible for many patients worldwide. One potential barrier to making medicines available to all is the cost of product registration, the fees for regulatory review and licensing for the sale of medicines beyond the cost of clinical trials, if needed.

**Methods and findings:**

We performed a cross-sectional analysis of pharmaceutical registration fees in low-, middle-, and high-income countries. We collected data on market authorization fees for new chemical entities and for generic drugs in 95 countries. We calculated measures of registration fee size relative to population, gross domestic product (GDP), and total health spending in each country. Each of the 95 countries had a fee for registering new chemical entities. On average, the ratio of registration fees to GDP was highest in Europe and North America and lowest in South and Central America. Across individual countries, the level of registration fees was positively correlated with GDP and total health spending, with relatively few outliers.

**Discussion:**

We find that, generally speaking, the regulatory fees charged by medicines regulatory authorities are roughly proportional to the market size in their jurisdictions. The data therefore do not support the hypothesis that regulatory fees are a barrier to market entry in most countries.

## Introduction

Ensuring equitable and affordable global access to medicines is one of the greatest public health challenges of our time. Advances in pharmaceuticals offer improved health outcomes for a wide range of illnesses, yet medicines are often inaccessible for many patients worldwide.[1] One potential barrier to making medicines available to all is the cost of product registration, the fees for regulatory review and licensing for the sale of medicines beyond the cost of clinical trials, if required. As registration fees are now the norm among regulatory authorities worldwide, there may be insufficient market opportunities for manufacturers of some medicines to make their products available in all markets, particularly in lower-income countries.

The purpose of this study was to assess new drug application fees in relation to market potential for medicines in high, middle, and low-income countries. We test the hypothesis that these fees are related to national income and identify countries for which application fees might pose a barrier to market entry. Kaplan and Laing addressed similar questions using 2002 data for 34 countries and found a positive correlation between incomes and registration fees.[2] In this paper, we update and extend that work with more recent data for a larger set of countries.

## Methods

This is a cross-sectional study of registration fees charged by regulatory authorities. During May through July 2016, we sought published regulatory fee information from any nation-state or supranational entity with a medicines regulatory agency. We found published cost information from websites of 68 medicines regulatory agencies identified using a list of regulatory authorities maintained by the World Health Organization.[3] We also obtained a regulatory fees database from Clarivate Analytics (formerly Thompson-Reuters), which include information for 78 medicines regulatory agencies.[4] Combined this yielded a study sample of 95 countries plus an observation for the centralized procedure of the European Medicines Agency S1 Table.

We collected regulatory fees for new chemical entities (NCEs) and for generic market authorization, where such cost information was available. If a regulatory authority had different fees for international and domestic manufacturers, we reported the fees for foreign products. For all countries with regulatory fee information, we gathered data on the national population, gross domestic product (GDP), health care spending, and currency conversion from the World Bank. We used GDP purchasing power parities for currency conversion and tested for sensitivity of results to the use of five-year average exchange rate conversions.

We calculated several measures of relative registration fee size: nominal registration fees in US dollars, registration fees per million residents, the ratio of registration fees per billion US dollars of GDP, and the ratio of registration fees per billion US dollars of health spending. We report descriptive statistics by region. We also plot registration fees in relation to GDP and health spending measured in terms of levels and natural logarithms per capita. We use linear regressions to measure correlations between registration fees and measures of the sizes of economies and health spending levels.

## Findings

### New chemical entities

Each of the 95 countries for which data were available had a fee for registering new chemical entities. These fees ranged from the equivalent of under US$10 in Bhutan and Nepal to over US$2 million in the USA (for a comprehensive fee that covers activities that may require additional fees in other countries). The interquartile range of fees was from US$1,080 to US$22,402.

The ratio of NCE registration fees per billion US dollars of GDP ranged from a low of essentially zero in Nepal to over 2,000 in Iceland. The interquartile range for the ratio of NCE registration fees per billion US dollars of GDP was 4.1 to 68.1. On average, the ratio of fees to GDP was highest in Europe and North America and lowest in Latin America–see Table 1. Registration fees relative to billions of US dollars in health expenditures varied as significantly as fees relative to GDP per capita and also resulted in the same ranking by region.

**Table 1:** Average ratio of new chemical entities (NCE) registration fees to billions of US dollars in gross domestic product (GDP) and total health expenditure, by region, with and without population weights.

	Average Ratio: NCE fee / US$US-billions of GDP	Population-weighted Average Ratio: NCE fee /US$US-billions of GDP	Average Ratio: NCE fee / US-billions of total health expenditure	Population-weighted Average Ratio: NCE fee / US-billions of total health expenditure
Africa	28.3	14.1	453.7	263.5
Arab States	12.1	6.4	189.5	112.0
Asia & Pacific	43.4	6.5	525.9	90.1
Commonwealth of Independent States	26.8	8.7	443.3	141.4
European Medicines Agency (EMA)*	18.6	18.6	199.6	199.6
Europe*	127.5	30.7	1,638.3	368.1
North America	126.6	116.7	1,159.6	883.9
South and Central America	7.9	12.1	115.7	160.2

* EMA figure represents the registration fee for EMA, divided by the entire population of member states. European region figures reflect the average of those countries for which national registration fees were available.

**Fig 1:**
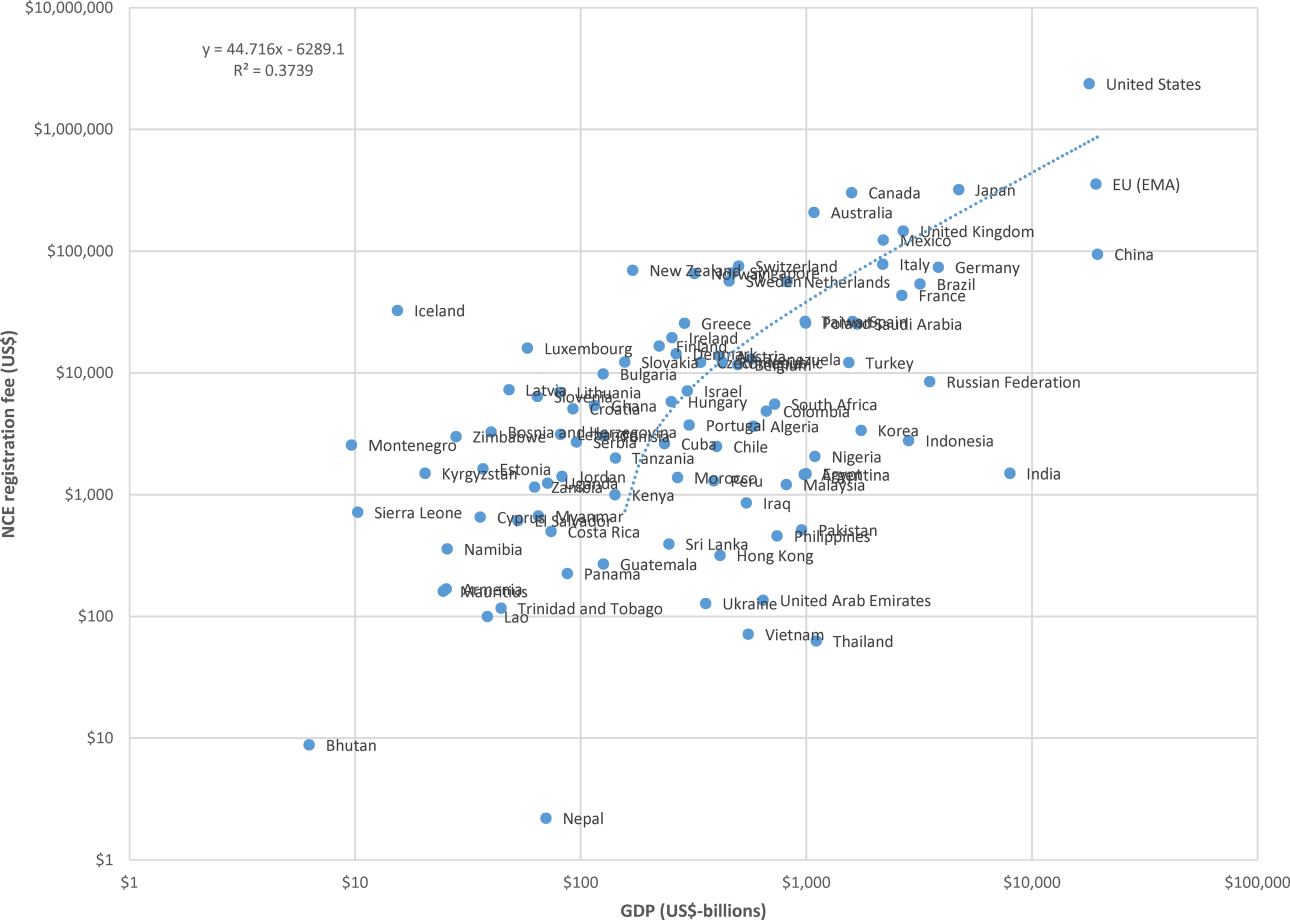
Plot of new chemical entity (NCE) registration fees to billions of US dollars in gross domestic product (GDP), logarithmic scale.

**Fig 2:**
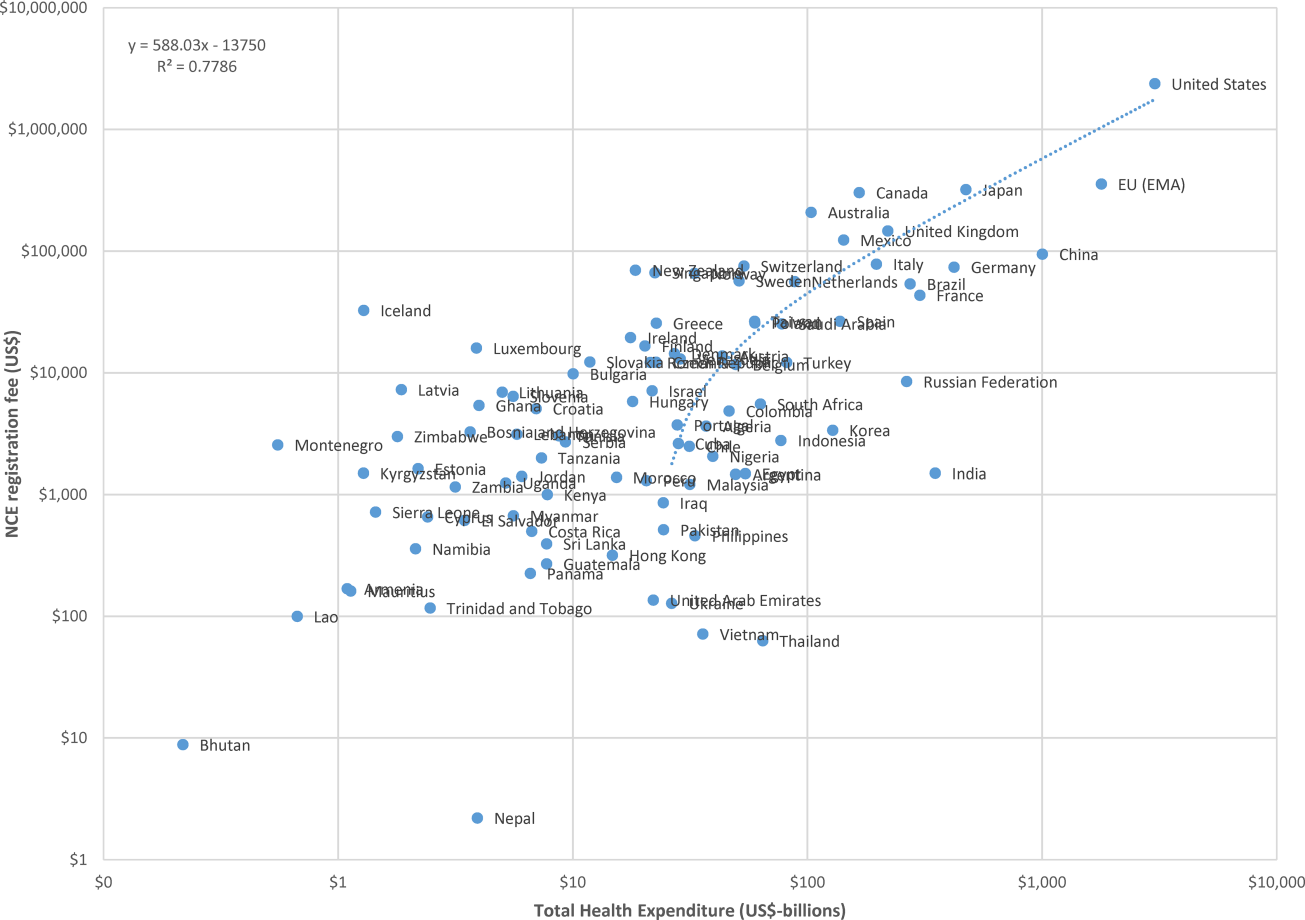
Plot of new chemical entity (NCE) registration fees to billions of US dollars in total health expenditure, logarithmic scale.

The data for NCE registration fees and billions of US dollars in GDP are plotted, using a logarithmic scale, in Fig 1. A linear regression of registration fees against national GDP results indicates that, on average across countries, product registration fees increase by approximately US$44 per billion US dollars of national GDP. Similar results are found for the relationship between NCE registration fees and billions of US dollars in national health expenditure, illustrated in Fig 2, which indicates that NCE registration fees increase by approximately US$588 per billion US dollars in total national health expenditure in countries.

**Fig 3:**
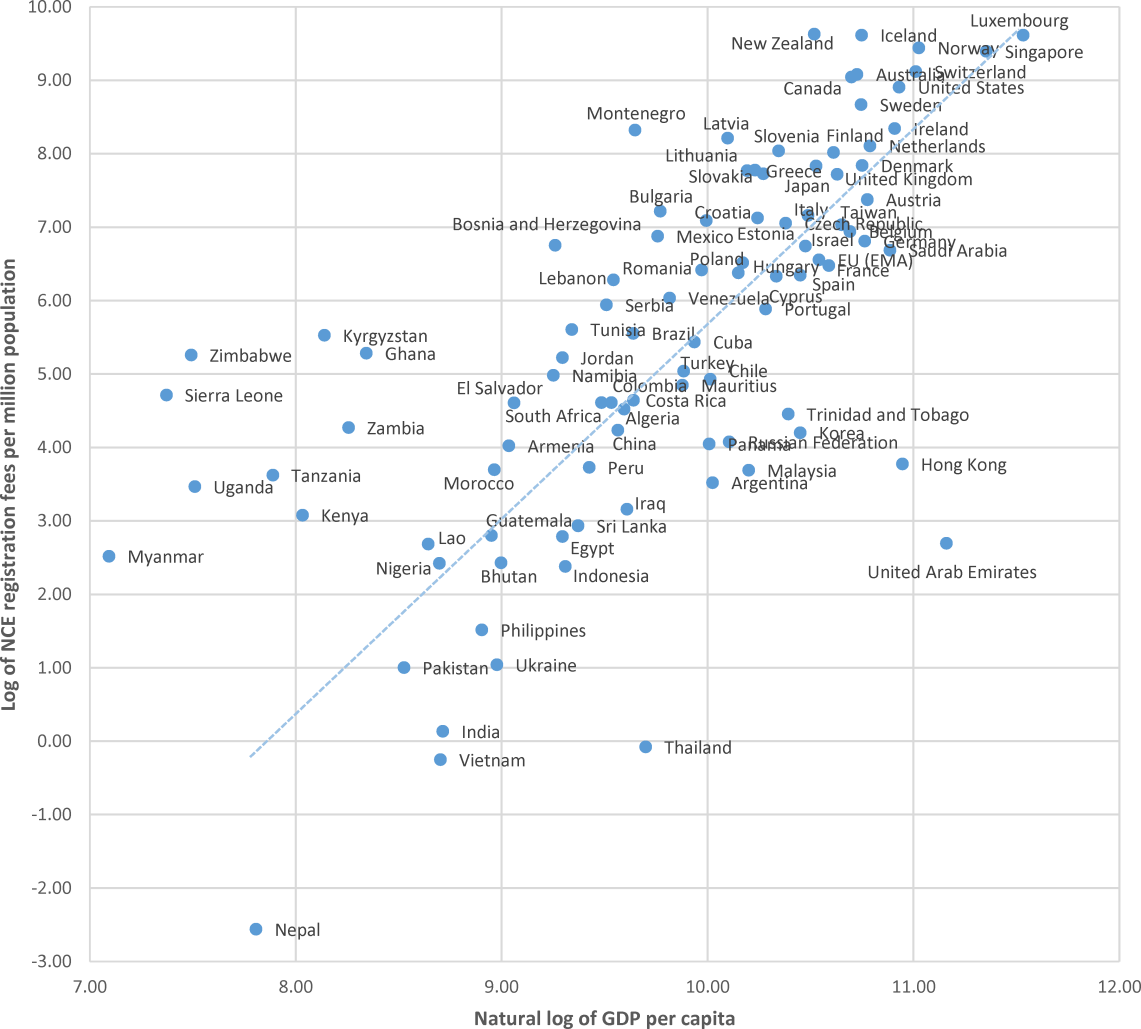
Plot of the natural log of new chemical entity (NCE) registration fees per million population to the natural log of US dollars of gross domestic product (GDP) per capita, with linear relationship among all countries except low-income outliers.

Because outliers in terms of overall size of the economy can have a pronounced affect the regression results pertaining to levels of GDP, we present a plot of the natural log of NCE registration fees per million population to the natural log of US dollars of GDP per capita in Fig 3. This standardizes both axes for population size, illustrating the relationship between registration costs per population and average incomes per population. Data illustrated in this way still shows a relationship between registration fees and income levels; however, it more clearly identifies a relatively small number of countries in which average registration fees may represent a high cost relative to average ability to pay, specifically the nine countries above the regression line at lower incomes per capita.

### Generic drugs

We were able to find published information on generic drug registration fees for 31 countries. These fees ranged from the equivalent of under US$100 in Armenia to approximately $80,000 in Australia and China. The interquartile range of fees was from US$2,635 to US$16,396.

**Fig 4:**
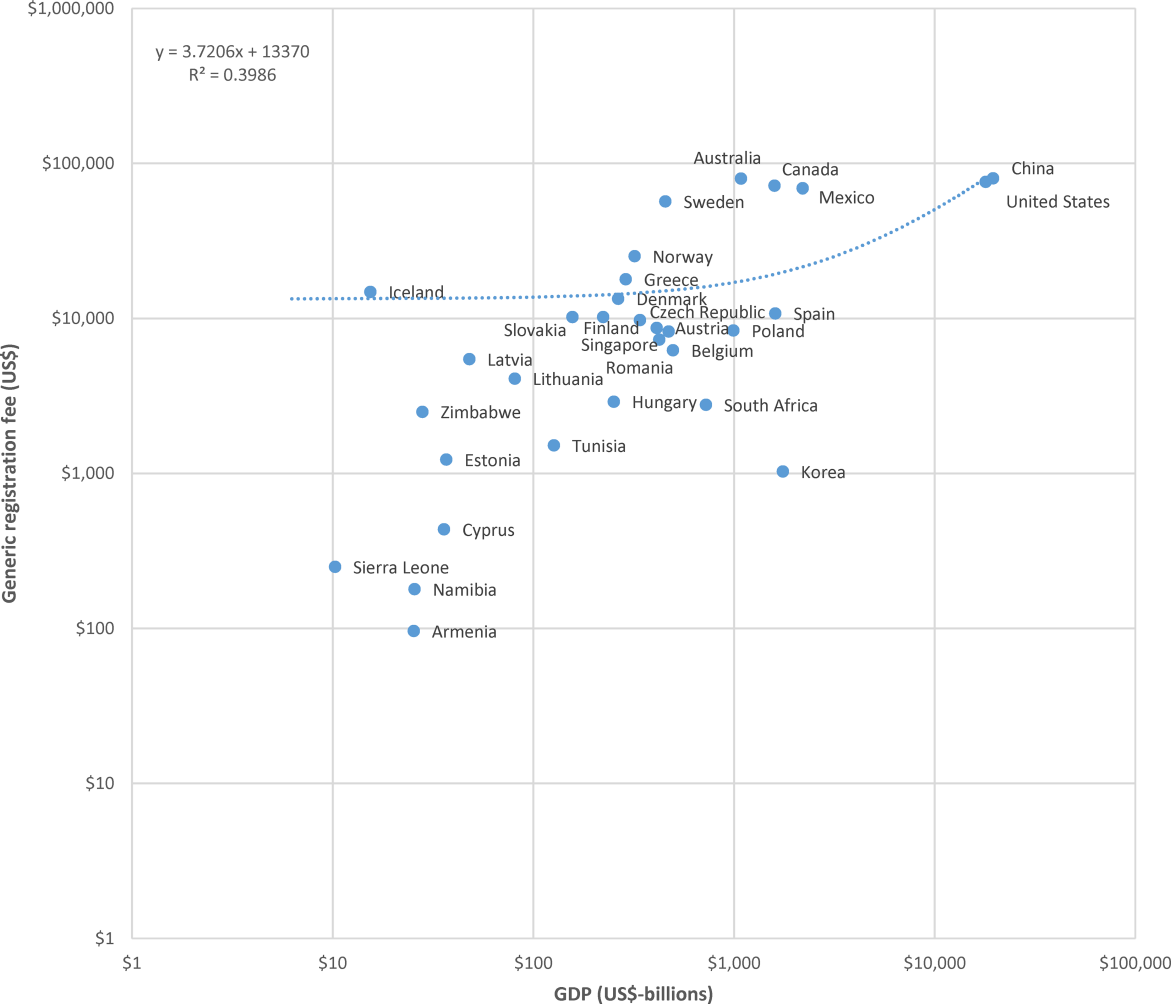
Plot of generic drug registration fees to billions of US dollars in gross domestic product (GDP), logarithmic scale.

**Fig 5:**
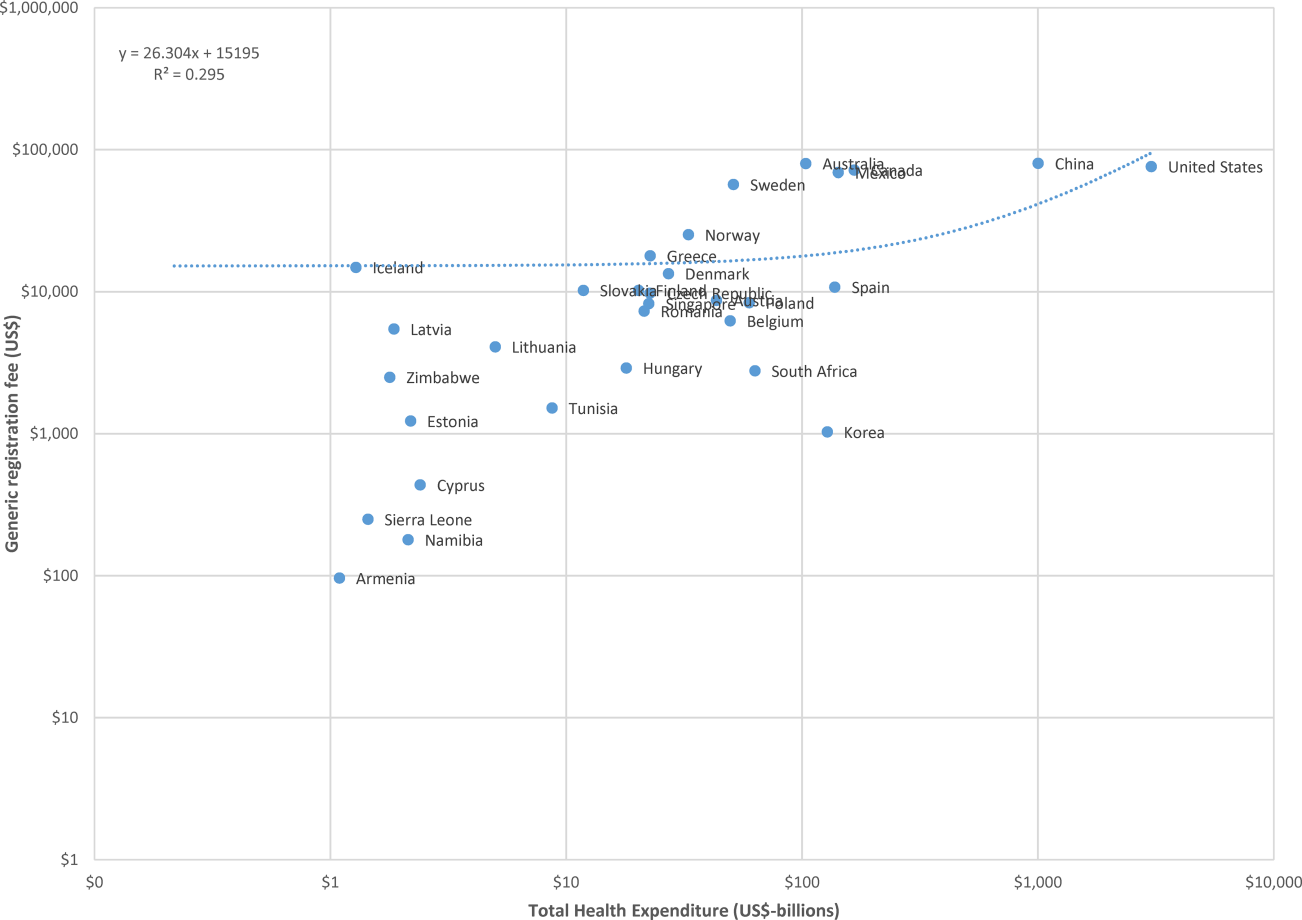
Plot of generic drug registration fees to billions of US dollars in total health expenditure, logarithmic scale.

The data for generic registration fees and billions of US dollars in GDP are plotted, using a logarithmic scale, in Fig 4. A linear regression of registration fees against national GDP results indicates that, on average across countries, generic product registration fees increase by approximately US$3.72 per billion US dollars of national GDP. Similar results are found for the relationship between generic registration fees and billions of US dollars in national health expenditure, illustrated in Fig 5.

**Fig 6:**
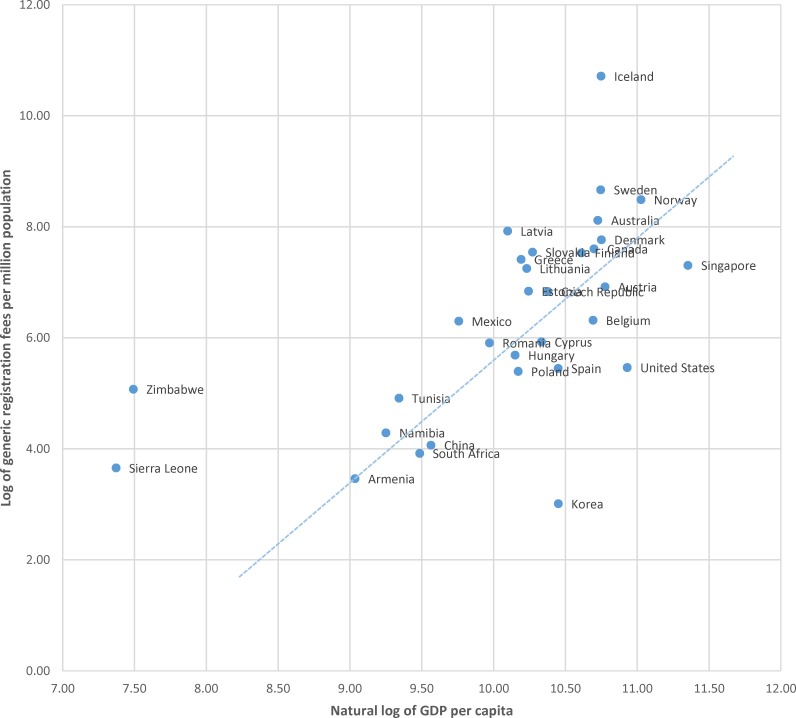
Plot of the natural log of generic drug registration fees per million population to the natural log of US dollars of gross domestic product (GDP) per capita, with linear relationship among all countries except low-income outliers.

Finally, Fig 6 plots of the natural log of generic registration fees per million population and the natural log of US dollars of GDP per capita. Shown in this way, the data illustrate that the generic registration fees in Zimbabwe and Sierra Leone may represent a high cost per population served given the average income per capita.

## Discussion

We find that, generally speaking, the regulatory fees charged by medicines regulatory authorities are roughly proportional to the market size in their jurisdictions. For most countries, therefore, the data do not support the hypothesis that regulatory fees are a barrier to market entry for manufacturers. This finding is generally consistent with the findings of Kaplan and Laing, who conclude that regulatory fees in some low-income countries could be increased without providing an undue disincentive to manufacturers[2].

There are, however, some exceptions insofar as regulatory fees are higher relative to incomes and health system size in a few low-income African and Asian countries. Although the absolute value of the fees in question are low, they may nevertheless be significant relative to the potential pharmaceutical market size. For example, the generic drug registration fee in the USA would have to equal $4.2 million in order to represent the same proportion of national income as the generic registration fee in Zimbabwe represents, even though fee in Zimbabwe may appear low in absolute value ($2,500). Case studies may help to determine whether the nominally-low but relatively-high fees in such outlying countries would represent a barrier to market entry.

Though our study suggests the monetary cost of entry is not likely a barrier to launching products in most markets, it is important to note that the financial cost is but one dimension of product registration processes. Other important considerations are the requirements, timelines, and consistency of regulatory processes. Ahonkhai and colleagues recently documented significant variations and delays in the time to final approval by regulatory agencies in Sub-Saharan Africa.[5] They argue that such barriers to timely product entry could be reduced through regulatory systems optimization, including relying on findings from reviews and inspections performed by trusted regulatory authorities, including WHO prequalification activities, to inform their own national regulatory decisions.

Our study is not without limitations. We have only assessed registration fees themselves and not issues concerning the timelines of regulatory assessments, which were most readily available on regulatory authority websites and the Clarivate Analytics database. Furthermore, we have assessed only the primary registration fees. In some countries, like the USA, these are relatively comprehensive fees covering a range of regulatory activities in addition to registration itself. In other countries, there can be several associated fees regarding product development for market entry, product registration, and post-registration vigilance activities that may vary by product type and manufacturer. We were able to find data on annual renewal fees for 40 countries; however, analyses of those data produced no statistically significant correlations between fees and measures of potential market size (GDP and health expenditures), and no notable outliers in terms of fee levels relative to measures of potential market size.

Medicines regulatory authorities must balance a variety of demands, including responsibility for public safety and the desire to ensure timely market access for products meeting standards of quality, safety, and efficacy. This can be a difficult task, especially for countries with limited resources.[6, 7] Registration fees may help offset some of the costs of regulation, and may assist agencies to strengthen their regulatory capacity; however, overreliance on fees to fund regulatory operations may impede some firms from entering small markets. Striking a balance is an important goal for global public health, one that may be aided through continued international cooperation among regulatory authorities, including WHO prequalification activities, and greater reliance on the resulting global science whenever appropriate, especially regarding inspection activities.

## Supporting information

S1 Table Registration fees for new chemical entities and generic drugs, US dollars. Source: Authors abstraction of data from websites of 68 medicines regulatory agencies combined with information provided by Clarivate Analytics (formerly Thompson-Reuters).
(DOCX)
