# Supplementary material for: The cost of entry: An analysis of pharmaceutical registration fees in low-, middle-, and high-income countries
Source: PLoS One. 2017 Aug 15;12(8):e0182742. doi: 10.1371/journal.pone.0182742 (PMC5557367; doi:10.1371/journal.pone.0182742)
Supplement: S1 Table — Source: Authors abstraction of data from websites of 68 medicines regulatory agencies combined with information provided by Clarivate Analytics (formerly Thompson-Reuters). (DOCX) [file pone.0182742.s001.docx]

The cost of entry: an analysis of pharmaceutical registration fees in low-, middle-, and high-income countries

##### Supplementary data

##### Registration fees for new chemical entities and generic drugs, US dollars. Source: Authors abstraction of data from websites of 68 medicines regulatory agencies combined with information provided by Clarivate Analytics (formerly Thompson-Reuters).

| Country | Currency | Application fee- new chemical entity | Application fee- generic | Annual registration fee (renewal of authorization fee) |
| --- | --- | --- | --- | --- |
| Algeria | USD | $3,649 |  | $365 |
| Argentina | USD | $1,468 |  | $1,114 |
| Armenia | USD | $168 | $96 |  |
| Australia | USD | $208,905 | $79,829 |  |
| Austria | USD | $13,765 | $8,704 |  |
| Belgium | USD | $11,701 | $6,240 | $2,925 |
| Bhutan | USD | $9 |  | $5 |
| Bosnia and Herzegovina | USD | $3,272 |  | $1,963 |
| Brazil | USD | $53,776 |  |  |
| Bulgaria | USD | $9,816 |  | $4,908 |
| Canada | USD | $303,956 | $71,853 |  |
| Chile | USD | $2,488 |  | $312 |
| China | USD | $94,750 | $80,088 | $7,243 |
| Colombia | USD | $4,850 |  |  |
| Costa Rica | USD | $500 |  |  |
| Croatia | USD | $5,084 |  | $3,389 |
| Cuba | USD | $2,620 |  |  |
| Cyprus | USD | $655 | $436 | $218 |
| Czech Republic | USD | $12,231 | $9,785 |  |
| Denmark | USD | $14,397 | $13,424 | $1,613 |
| Egypt | USD | $1,487 |  |  |
| El Salvador | USD | $615 |  |  |
| EU (EMA) | USD | $356,869 |  | $128,002 |
| Estonia | USD | $1,632 | $1,226 | $818 |
| Finland | USD | $16,640 | $10,240 | $1,728 |
| France | USD | $43,521 |  |  |
| Germany | USD | $73,985 |  | $16,128 |
| Ghana | USD | $5,400 |  |  |
| Greece | USD | $25,600 | $17,920 | $6,400 |
| Guatemala | USD | $270 |  | $96 |
| Hong Kong | USD | $318 |  |  |
| Hungary | USD | $5,810 | $2,905 | $2,905 |
| Iceland | USD | $32,685 | $14,872 | $245 |
| India | USD | $1,500 |  |  |
| Indonesia | USD | $2,784 |  |  |
| Iraq | USD | $857 |  |  |
| Ireland | USD | $19,470 |  |  |
| Israel | USD | $7,124 |  |  |
| Italy | USD | $78,398 |  |  |
| Japan | USD | $320,839 |  |  |
| Jordan | USD | $1,408 |  |  |
| Kenya | USD | $1,000 |  | $500 |
| Korea | USD | $3,379 | $1,028 |  |
| Kyrgyzstan | USD | $1,500 |  |  |
| Lao | USD | $100 |  |  |
| Latvia | USD | $7,285 | $5,464 |  |
| Lebanon | USD | $3,134 |  | $2,239 |
| Lithuania | USD | $6,929 | $4,095 | $1,335 |
| Luxembourg | USD | $16,000 |  | $128 |
| Malaysia | USD | $1,214 |  |  |
| Mauritius | USD | $161 |  | $64 |
| Mexico | USD | $123,475 | $69,056 |  |
| Montenegro | USD | $2,560 |  |  |
| Morocco | USD | $1,386 |  | $23 |
| Myanmar | USD | $671 |  | $671 |
| Namibia | USD | $359 | $180 | $108 |
| Nepal | USD | $2 |  | $1 |
| Netherlands | USD | $56,193 |  |  |
| New Zealand | USD | $69,882 |  |  |
| Nigeria | USD | $2,057 |  |  |
| Norway | USD | $65,534 | $25,266 | $7,106 |
| Pakistan | USD | $515 |  | $206 |
| Panama | USD | $225 |  |  |
| Peru | USD | $1,305 |  |  |
| Philippines | USD | $459 |  |  |
| Poland | USD | $25,760 | $8,372 |  |
| Portugal | USD | $3,732 |  | $2,252 |
| Romania | USD | $12,160 | $7,296 |  |
| Russian Federation | USD | $8,491 |  |  |
| Saudi Arabia | USD | $25,333 |  |  |
| Serbia | USD | $2,705 |  |  |
| Sierra Leone | USD | $720 | $250 | $520 |
| Singapore | USD | $66,595 | $8,241 |  |
| Slovakia | USD | $12,288 | $10,240 | $6,400 |
| Slovenia | USD | $6,400 |  | $1,920 |
| South Africa | USD | $5,540 | $2,770 |  |
| Spain | USD | $26,540 | $10,796 | $2,998 |
| Sri Lanka | USD | $395 |  |  |
| Sweden | USD | $57,016 | $57,016 | $6,557 |
| Switzerland | USD | $75,575 |  |  |
| Taiwan | USD | $26,546 |  |  |
| Tanzania | USD | $2,000 |  |  |
| Thailand | USD | $63 |  |  |
| Trinidad and Tobago | USD | $117 |  |  |
| Tunisia | USD | $3,029 | $1,514 |  |
| Turkey | USD | $12,156 |  |  |
| Uganda | USD | $1,250 |  | $500 |
| Ukraine | USD | $128 |  |  |
| United Arab Emirates | USD | $136 |  |  |
| United Kingdom | USD | $146,802 |  |  |
| United States | USD | $2,374,300 | $76,030 | $114,450 |
| Venezuela | USD | $13,017 |  | $4,881 |
| Vietnam | USD | $71 |  |  |
| Zambia | USD | $1,159 |  |  |
| Zimbabwe | USD | $3,000 | $2,500 | $500 |
